# Non-Pregnant and Pregnant Women’s Femininity Preferences in Male Faces: Tests Based on Within- and Between-Sex Sexual Dimorphism Facial Manipulations

**DOI:** 10.1007/s10508-020-01868-8

**Published:** 2021-01-04

**Authors:** Fangfang Wen, Bin Zuo, Yang Wang, Shuhan Ma, Shijie Song, Hongxia Zhang

**Affiliations:** 1 School of Psychology, Center for Studies of Social Psychology, Central China Normal University, Key Laboratory of Adolescent Cyberpsychology and Behavior, Ministry of Education, Wuhan, 430079 China; 2 Xiantao First People’s Hospital of Hubei Province, Xiantao, China

**Keywords:** Facial attractiveness, Sexual dimorphism, Pregnancy, Facial manipulation

## Abstract

Past research on women’s preferences for male facial masculinity in Western cultures has produced inconsistent results. Some inconsistency may be related to the use of different facial stimulus manipulations (e.g., between-sex sexual dimorphic facial manipulation or within-sex sexual dimorphic facial manipulation) that do not perfectly avoid non-facial cues, and pregnancy status may also influence women’s face preferences. We therefore recruited pregnant and nonpregnant Chinese women and manipulated the sexual dimorphism of male facial stimuli to explore the influences of manipulation methods, non-facial cues, and pregnancy status on face preferences. Results showed that: (1) in contrast with a general masculinity preference observed in Western cultures, both pregnant and nonpregnant Chinese women preferred feminized and neutral male faces generally; (2) pregnant women’s preference for feminized male faces was stable across manipulation methods, while nonpregnant women preferred feminized male faces except under between-sex sexual dimorphism manipulation; and (3) manipulation methods, rather than non-facial cues, influenced participants’ face preferences. Specifically, women showed the strongest preferences for femininity when face stimuli were manipulated by within-sex sexual dimorphic facial manipulation, followed by unmanipulated faces and between-sex sexual dimorphic facial manipulation. This effect was stronger for nonpregnant women in the unmanipulated condition and for pregnant women in the between-sex sexual dimorphic facial manipulation. This research provides empirical evidence of women’s preferences for sexual dimorphism in male faces in a non-Western culture, as well as the effects of facial manipulation methods, pregnancy status, and the interactions between these factors.

**Electronic supplementary material:**

The online version of this article (10.1007/s10508-020-01868-8) contains supplementary material, which is available to authorized users.

## Introduction

Research suggests that facial attractiveness has important consequences for individuals’ well-being and social interactions. For example, facial attractiveness is closely associated with individuals’ mate selection, the likelihood of obtaining employment, and health status (Buckingham et al., 2006), and it is implicated in evolutionary and anthropological explanations of social interaction (Skomina, Verdenik, & Hren, 2020). One important indicator of facial attractiveness is sexual dimorphism, in this case the different characteristics in male and female faces (Burriss, Urszula, & Lyons, 2014; Enquist, Ghirlanda, Lundqvist, & Wachtmeister, 2002; Gangestad & Scheyd, 2005; Zuo, Wen, & Wu, 2019). Recently, the relationship between sexually dimorphic faces and the perception of facial attractiveness has been broadly examined, especially regarding females’ preferences for male faces (DeBruine, Jones, Smith, & Little, 2010; DeBruine et al., 2006; Rennels, Bronstad, & Langlois, 2008; Wen & Zuo, 2012). However, there is a lack of consensus in the research on whether women prefer masculinized or feminized male faces.

Previous literature suggests a relationship between women’s preference for sexual dimorphism in males faces and a high possibility of fertility, e.g., being of childbearing age (Little, DeBruine, & Jones, 2010) or in the late follicular phase (ovulatory) phase of the menstrual cycle (Little, Jones, & DeBruine, 2008). However, more recent studies with larger sample sizes and more precise measures of ovulatory cycle variation have found little evidence of such relationships (e.g., Jones et al., 2018; Stern, Arslan, Gerlach, & Penke, 2019), and thus, it is unclear whether women’s hormonal levels influence their face preferences. Pregnancy brings a series of tremendous changes of hormonal levels within women’s bodies (Robinson & Klein, 2012) and could therefore shed further light on whether and how women’s preferences are influenced by hormonal levels. Past research that explored a possible influence of pregnancy status or breastfeeding in women’s preferences for masculinity in traits such as voices (Apicella, Feinberg, & Marlowe, 2007; Shirazi, Puts, & Escasa-Dorne, 2018) and faces (Escasa-Dorne, Manlove, & Gray, 2017) found that women with productive potential would prefer masculine traits, while those who recently give birth to babies like feminine male faces was more.

Different face morphing methods also influence women’s preferences (DeBruine et al., 2006, 2010; Rennels et al., 2008). Therefore, it is worth exploring whether and how face morphing methods interact with pregnancy status to influence women’s face preferences. Therefore, the present study focused on comparing pregnant and nonpregnant women’s preferences for sexually dimorphic male faces manipulated with different morphing technologies.

### Evolutionary Psychology and Women’s Preferences for Male Faces

The good genes theory of sexual selection (Fink & Penton-Voak, 2002) and the trade-off model of strategic pluralism (Gangestad & Simpson, 2000) explain women’s face preferences from evolutionary perspectives. According to the good genes theory, faces suggestive of healthy genes and preeminent reproductivity are more attractive due to their heredity advantages (Fink & Penton-Voak, 2002; Scott et al., 2014). Indeed, masculine cues in male faces are positively correlated with levels of testosterone (Penton-Voak & Chen, 2004; Roney, Hanson, Durante, & Maestripieri, 2006), and testosterone-dependent traits have been suggested to be reliable indicators of genes that contribute to healthy immune system function (Rhodes, 2006; Thornhill & Gangestad, 1999, 2006; but see Zaidi et al., 2018). Therefore, women are expected to prefer masculinized male faces (Gangestad & Scheyd, 2005), and most research supports this expectation (e.g., DeBruine et al., 2006, 2010).

However, some studies have found that women prefer feminized male faces (DeBruine et al., 2006; Jones et al., 2005; Little & Mannion, 2006; Rhodes, Hickford, & Jeffery, 2000). The trade-off model of strategic pluralism suggests the preference bias for masculinized or feminized male faces reflects a trade-off between healthy genes and potential parental investment (Gangestad & Simpson, 2000). Men with more feminine faces are perceived as possessing more pro-social personalities and accepting more responsibility for their offspring (Perrett et al., 1998). They also have longer romantic relationships and relatively stable marriages (Rhodes, Simmons, & Peters, 2005). In contrast, men with more masculine faces are frequently perceived as being less willing to make commitments in a relationship, having a higher infidelity rate, and being irresponsible fathers who spend less time and share fewer resources in the raising of children (Boothroyd, Jones, Burt, & Perrett, 2007; Gildersleeve, Haselton, & Fales, 2014; Kruger, 2006; Little, Jones, & DeBruine, 2011; Oosterhof & Todorov, 2008; Perrett et al., 1998).

### Women’s Fertility/Hormonal Differences and Masculinity Preferences

Women’s fertility and hormonal differences may influence their trade-off between good genes and parental investment (Welling, DeBruine, Little, & Jones, 2009; Welling et al., 2007; Zietsch, Lee, Sherlock, & Jern, 2015) and thus influence their preference for sexual dimorphism in male faces (Gangestad & Thornhill, 2008; Jones, Obregón, Kelly, & Branigan, 2008). Compared to women in late puberty with high fertility, pre-pubescent and post-menopausal women showed a lower preference for masculinized male faces (Little et al., 2010; Sacco, Jones, DeBruine, & Hugenberg, 2012). Furthermore, some researchers found that only women in a period of high-level fertility preferred masculine men as mating partners (Gangestad & Thornhill, 2008; Gildersleeve et al., 2014; Little, Connely, Feinberg, Jones, & Roberts, 2011). However, some recent studies with larger samples and hormonal concentrations rather than self-report for measuring cycle phase showed no evidence for women’s stronger preference for masculinity when fertile (e.g., DeBruine, Hahn, & Jones, 2019; Jones et al., 2018; Stern et al., 2019). Considering the possible influence of women’s fertility on their preference for sexually dimorphic male faces, we sought to investigate how the biological state of pregnancy influences women’s preferences.

Pregnancy is a time of tremendous hormonal changes, especially in the levels of estrogen and progesterone, which may affect face preferences (Jones et al., 2008; Roney & Simmons, 2008; Theodoridou, Rowe, Penton-Voak, & Rogers, 2009; Welling et al., 2007, 2009). Perhaps due to an increase in progesterone (Dixson, 2009), pregnant women have a lower sex drive and showed a lower preference for masculine male faces (or sexy male faces) than feminine male faces (or friendly male faces) (Kościński, 2011). One study comparing adolescent girls, nonpregnant young women, pregnant young women, and middle-aged women showed that pregnant women and menopausal women exhibited a similar pattern in face preferences (Kościński, 2011). This finding may be related to the fact that women and their babies are vulnerable during pregnancy and the next few years; this vulnerability could lead to women’s dependence on others and their need for care during pregnancy (Leifer, 1977). Therefore, because paternal investment may be more important than other factors in potential mates, and women’s preferences for highly masculine men (Dixson, Kennedy-Costantini, Lee, & Nelson, 2019) and/or masculinized faces (Cobey, Little, & Roberts, 2015; Limoncin et al., 2015) may decrease during pregnancy.

### Facial Manipulation Methods

There are two main manipulation methods for generating male faces with sexual dimorphism cues: the between-sex sexual dimorphism (BSSD) and within-sex sexual dimorphism (WSSD) facial manipulation methods (Perrett et al., 1998). BSSD facial manipulation creates a continuum along prototypes of the female face and the male face, and the prototypes are generated by averaging several male or female faces. Using BSSD facial manipulation, sexually dimorphic male faces are created by morphing the male prototype away or toward the shape of the female prototype (for more details, see the following Method sections: DeBruine et al., 2006; Perrett et al., 1998; Wen & Zuo, 2012). WSSD facial manipulation generates masculinized and feminized male faces directly by averaging several of the most masculine/feminine male faces (as rated by participants; Johnston, Hagel, Franklin, Fink, & Grammer, 2001; Wen & Zuo, 2012).

The BSSD morphing method implies a one-dimensional model of facial sexual dimorphism, in which faces lie along a single male–female continuum. Moreover, BSSD facial manipulation is consistent with the traditional sex-role hypothesis, in which the sex-role is a one-dimensional structure with extreme masculine and feminine traits at two opposing ends (Cai & Yang, 2002). Conversely, WSSD facial manipulation regards masculinized and feminized male faces as separate dimensions of facial shape and is therefore consistent with the sex-role theory proposed by Bem (1974), which also treats masculinity and femininity as independent dimensions (Xu et al., 2010).

From the different logic underlying the BSSD and WSSD morphing methods, we predicted that more feminine features might be contained in facial stimuli produced via BSSD facial manipulation. Meanwhile, facial stimuli produced via WSSD facial manipulation only reflects the variance of faces within each gender. The subtle difference between face stimuli of BSSD and WSSD facial manipulation could become obvious for those who are sensitive to feminine characteristics. Pregnant women, for example, might have this sensitivity as they have to pay more attention to the parental investment of potential mates (Leifer, 1977). Thus, facial manipulation methods could interact with pregnancy status and jointly influence women’s preference for male faces.

### The Present Study

The current study explored the influence of pregnancy and facial manipulation methods on women’s preference for sexually dimorphic male faces in a Chinese sample. Compared to Western culture, Chinese culture stresses pro-social behavior for building and maintaining harmonious interpersonal relationships (Ma, Tunney, & Ferguson, 2017; Wang & Cui, 2007). In addition, masculinity in China is centered on internal characteristics rather than physical appearances (Gutierrez et al., 2020). Therefore, Chinese culture might be more relaxed regarding the masculine appearance of males. Consistent with previous findings (Wen & Zuo, 2012), we expected a greater preference for feminized male faces in Chinese participants compared to Western participants.

Based on previous literature, we also expected that pregnant women would show a stronger preference than nonpregnant women for feminized male faces. As previously discussed, pregnant women are more sensitive than nonpregnant women to gender cues that imply a paternal investment (Leifer, 1977). This sensitivity may lead to different preferences when masculinized and feminized male faces are morphed using the single dimension of BSSD facial manipulation, rather than two-dimensional WSSD facial manipulation. Since pregnant women have shown a lower preference for masculinized male faces (Cobey et al., 2015; Limoncin et al., 2015), we predicted that pregnant women would show a stronger preference for feminized male faces, especially in the BSSD facial manipulation condition. However, we predicted that no difference would be found in the WSSD facial manipulation condition between pregnant and nonpregnant women.

Furthermore, the existence of non-facial cues is always examined in research on sexual dimorphism face preferences. DeBruine et al.’s (2010) research showed that women’s preference patterns for male faces were consistent between two morphing technologies in the masked condition but differed in the unmasked conditions. In addition, DeBruine et al. demonstrated that controlling facial cues such as hairstyle in experiments would affect male facial masculinity. Therefore, we used both unmasked faces and masked faces in the current study, to explore whether the existence of non-facial cues would interact with pregnancy and facial manipulation methods to influence women’s face preferences. Adding this factor into our experiment not only helped to replicate previous findings, but also increased the reliability of our results.

## Method

### Participants

A total of 157 female participants were recruited from Wuhan, China, including 125 female college students (*M*_age_ = 19.90 years, *SD *= 0.70, ages ranging from 18 to 21) and 32 pregnant women (*M*_age_ = 27.66, *SD* = 4.60, ages ranging from 19 to 44). The student participants were recruited from the campus of a college, while the pregnant participants were recruited from a pregnancy care center that provides regular medical tests for pregnant women. Considering the influence of sexual orientation (Glassenberg, Feinberg, Jones, Little, & DeBruine, 2009) and contraception use on face preferences (Little, Burriss, Petrie, Jones, & Roberts, 2013), we tried to eliminate these factors using a self-report questionnaire. All participants reported themselves as being heterosexual and not using contraception. All pregnant participants reported the length of their pregnancies at the time of the experiment, which ranged from five to nine months. Two participants failed to complete all the trials due to restrictions in physical movement. However, considering the limited number of pregnant participants, they were still included in our final sample, and the trials they failed to complete were treated as missing data.

### Materials

Facial photographs were obtained from the database of a Chinese university graduate information registration system. All facial photographs were taken under standardized circumstances, with the same background and uniform luminance. As this registration system restrained the size of photographs uploaded, the materials in this experiment looked somewhat pixelated. However, all facial features and sexual dimorphism information in the faces could still be distinguished; we believe this confounding factor did not influence the direction of our results (see Supplemental Materials for the face manipulation check procedure). A total of 321 facial photographs were available, including 144 males and 177 females. After excluding people with eyeglasses, mustaches, jewelry, and non-neutral expressions, 64 original faces were selected (32 for each sex). These photographs were used to generate the prototype of male and female faces, which were used to create masculinized, feminized, and unaltered male composite faces in the BSSD and WSSD facial manipulation conditions.

#### BSSD Facial Manipulation

**Fig. 1:**
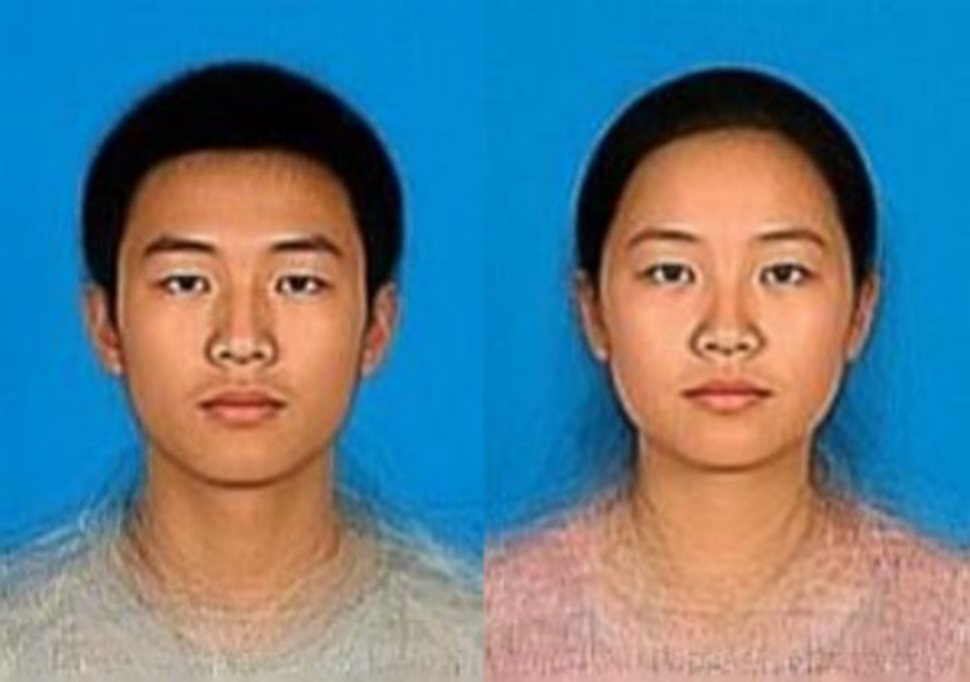
Face prototypes of both sexes

Face stimuli were generated followed procedures established by DeBruine et al. (2010) and Rennels et al. (2008). First, we used FantaMorph 4.0 software to generate facial prototypes from 32 photographs of both sexes. A total of 179 key points delineating the shape and contours of each face were marked, highlighting all recognizable features. Then, we averaged the key points for the original male and female faces separately to generate the facial prototype of each sex (Fig. 1). After generating the male and female facial prototypes, we morphed the male prototype to create the stimuli for the BSSD facial manipulation condition. BSSD male faces were manipulated according to Perrett et al.’s (1998) procedures to exaggerate or diminish the differences in features between the male and female prototypes to create their masculinized and feminized versions. To achieve this, we morphed the shape of the male prototype 50% toward or away from the female prototype, obtaining the masculinized male face and feminized male face, respectively (Perrett et al., 1998; Rhodes et al., 2000). This procedure was performed through DeBruine et al.’s website (www.faceresearch.org). Finally, we obtained the formal unmasked BSSD stimuli including a masculinized male face, an unaltered male composite face (the male prototype), and a feminized male face.

#### WSSD Facial Manipulation

A pilot study was conducted to generate photographs of perceived feminized and masculinized male faces for WSSD facial manipulation. A total of 60 facial photographs of men were selected from the original 144 photographs of men, with the exclusion criterion of extra facial decorations, e.g., beard or earrings, to prevent the influence of non-facial cues on ratings. A total of 26 self-reported heterosexual female undergraduates (*M*_age_ = 23.54, *SD* = 3.99) rated the perceived masculinity of the 60 male facial photographs on a 7-point Likert scale (1 = *lowest masculinity*, 7 = *highest masculinity*). The reliability (Cronbach’s *α*) between participants was 0.91, and the mean masculinity rating of all faces was 4.42, *CI* = [2.23, 5.81]. We then selected the 14 photographs with the highest masculinity ratings (23.5%, *M* = 4.37) and the 14 photographs with the lowest masculinity ratings (23.5%, *M* = 3.33) and averaged them to generate the masculinized and feminized male faces in the WSSD facial manipulation condition, respectively. The face morphing process also used FantaMorph 4.0 software that delineated and then averaged 179 key points extracted from each face. Finally, the within-sex unaltered male composite face was generated by averaging the within-sex masculinized and feminized male faces. As a result, we obtained the formal unmasked WSSD stimuli, including a masculinized male face, a feminized male face, and an unaltered male composite face.

#### Unmanipulated Faces Selection

The unmanipulated faces were also included as a facial manipulation condition and were used as a reference group to increase the ecological validity of the study (Wen & Zuo, 2012). Similar to prior research (Wen & Zuo, 2012), the stimuli in this condition were based on the results of the pilot study conducted for the WSSD facial manipulation and consisted of the three male photographs with the highest masculinity rating (*M* = 4.37, *SD* = 0.88), mean masculinity rating (*M* = 4.42, *SD* = 0.76), and lowest masculinity rating (*M* = 3.33, *SD* = 0.79), which were labeled the “most masculine face,” “most neutral face,” and “most feminine face,” respectively.

#### Non-Facial Cues Manipulation

**Fig. 2:**
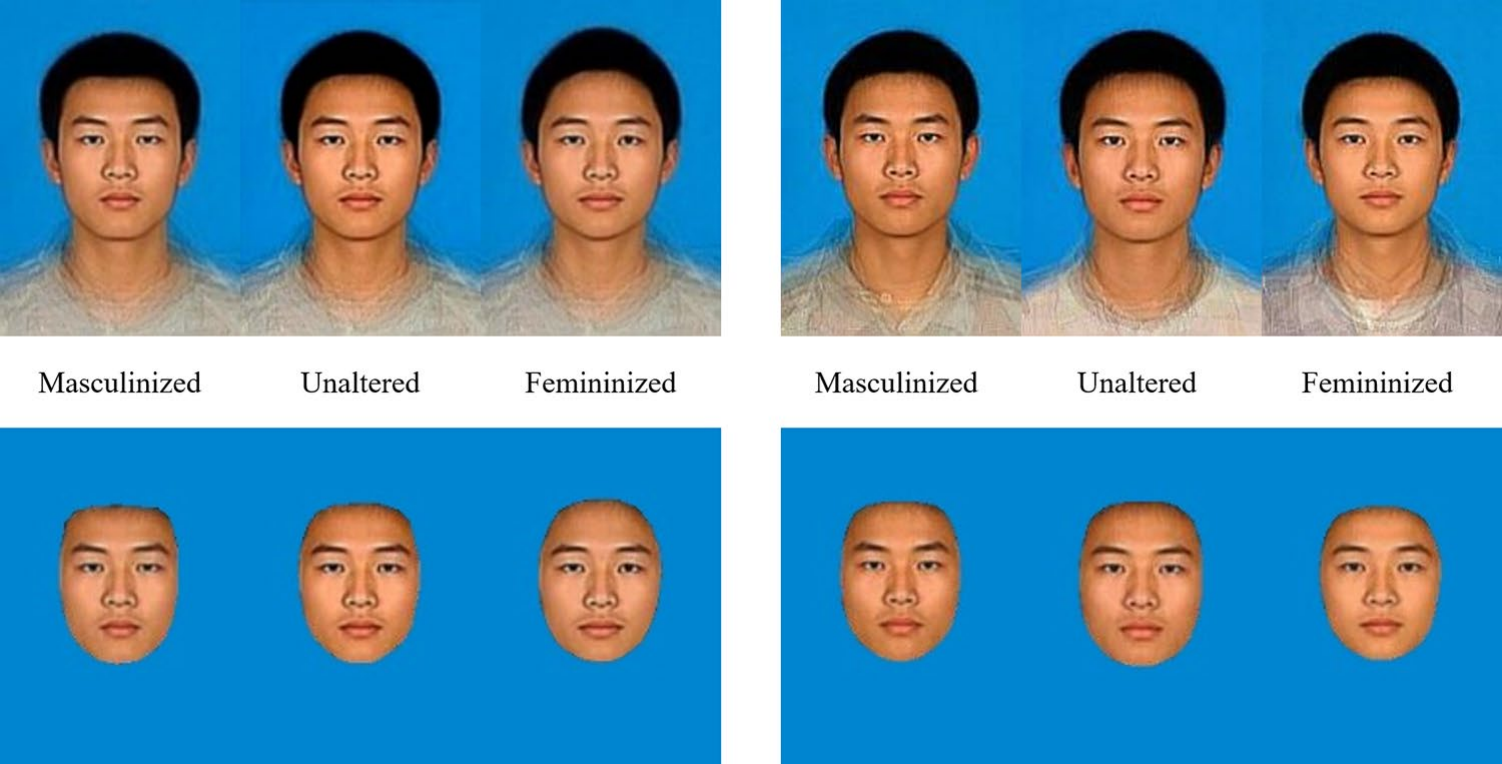
Formal face stimulus of BSSD and WSSD facial manipulation. The left panel shows faces morphed by the BSSD facial manipulation, while the right panel shows faces morphed by WSSD facial manipulation. The upper panel shows unmasked faces, while the bottom panel shows masked faces. The unmanipulated faces were not presented because of privacy considerations

Unmasked faces were the masculinized, unaltered, and feminized male composite faces generated by three facial manipulation conditions. The masked faces were obtained by removing all non-facial cues in the unmasked face stimulus, such as hair, neck, and clothes. Corresponding stimuli are illustrated in the bottom panel of Fig. 2. Finally, to exclude the potential influences of picture size and color, 18 formal face materials, standardized at 400 × 300 pixels with a blue background, were obtained.

### Design and Procedure

A 2 (participant type: nonpregnant women vs. pregnant women) × 2 (non-facial cue: masked vs. unmasked) × 3 (face manipulation method: BSSD vs. WSSD vs. unmanipulated face) mixed design was employed for this experiment. The participant type was a between-subject factor, while non-facial cue and face manipulation method were within-subject factors. The dependent variable was the participants’ preference for feminine or masculine male faces. Altogether, we generated six within-subject conditions: (1) masked and unmasked faces manipulated by BSSD facial manipulation, (2) masked and unmasked faces manipulated by WSSD facial manipulation, and (3) masked and unmasked unmanipulated faces conditions. In each within-subject condition, three faces were randomly paired and presented to participants, i.e., masculinized vs. unaltered (M–U) faces, masculinized vs. feminized (M–F) faces, and unaltered vs. feminized (U–F) faces. Therefore, this test presented 18 trials in random order; each trial included two faces and asked the participants to choose the more attractive one.

The same procedure for selecting face preferences was conducted for nonpregnant and pregnant participants, albeit through different platforms. For nonpregnant women, the formal experiment was employed in an online survey platform (www.wjx.cn), and participants who gave their informed consent were tested independently in a quiet room under the supervision of an experimenter.

**Fig. 3:**
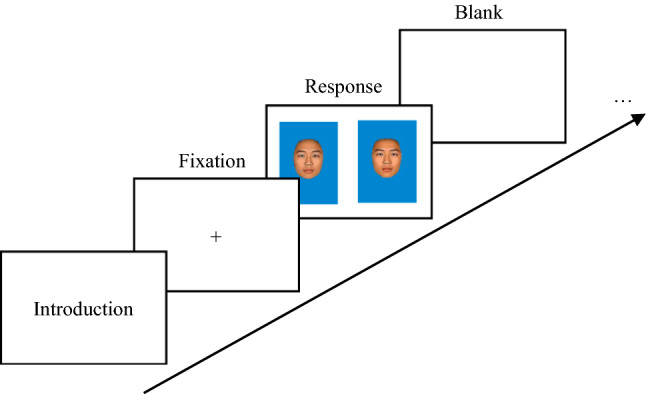
The procedure of each trial for pregnant participants

For pregnant women, however, who were recruited from and tested in a pregnancy care center, we used an E-prime program to collect the data more efficiently and conveniently. The E-prime program was conducted in Huawei Matebook 10 with a touch screen; pregnant women only needed to touch the more attractive face in each face pairing without having to slide the screen. Each trial began with a fixation presented for 500 ms; then, two balanced male faces were presented on both sides of the screen (Fig. 3). Participants were required to choose the more attractive face in each pairing, without time limits. A 500 ms inter-trial interval was presented with a blank screen.

### Analytical Approach

Prior to the formal data analysis, we created two binary variables for male facial masculinity and femininity and coded participants’ responses for each trial based on whether they indicated the corresponding preference. For the M–U faces trials, a neutral face preference was coded as a femininity preference. Similarly, for all U–F faces trials, a neutral face preference was coded as a masculinity preference. For all the M–F faces trials, the codes of participants’ responses were consistent with their choice for male facial masculinity or femininity.

A unified two-level binary logistic regression model was employed to analyze the data. This unified model allowed us to evaluate the effect of independent variables at both the measurement level (that is, within-subject variables: non-facial cues and the facial manipulation method) and the participant level (that is, between-subject variables: participant type and age). All nominal variables (i.e., non-facial cues, facial manipulation method, and participant type) were transformed into dummy variables. For the three conditions in the face manipulation method, two dummy variables were used to represent the BSSD and WSSD manipulation variables, while the unmanipulated facial condition was treated as the reference variable. In addition to the main effects, the interactions between participant type and facial manipulation method, and participant type and non-facial cue, were also included in the model to help determine how women’s pregnancy status interacted with the objective attributes of the male faces. Compared to the effect of independent variables or interactions, the binomial analysis examined the differences in women’s preferences for male faces more specifically.

## Results

The frequencies of participants’ face preferences are shown in Table 1. The one-sample binomial test revealed that except for nonpregnant women in the masked (*p* = .283) and unmasked (*p* = .721) BSSD facial manipulation conditions, and pregnant women in the unmasked BSSD facial manipulation condition (*p* = .472), participants generally exhibited a significant preference for feminized male faces (all *p* < .013).

**Table 1:** The frequency of male faces preference for nonpregnant and pregnant women under each facial manipulation method

Non-facial cue	Preference	Nonpregnant women (%)			Pregnant women (%)		
		Between-sex	Within-sex	Unmanipulated	Between-sex	Within-sex	Unmanipulated
Unmasked	Masculinity	52.0	16.8	38.4	40.6	21.9	21.9
	Femininity	48.0	83.2	61.6	56.3	71.9	71.9
Masked	Masculinity	55.2	16.0	28.0	21.9	21.9	18.8
	Femininity	44.8	84.0	72.0	78.1	71.9	78.1

Preference refers to participants’ faces preference in each condition; masculinity indicates the masculinized male face preference; femininity refers to the feminized male face preference. Between-sex, within-sex, and unmanipulated indicate three different facial manipulation methods of male faces

A further two-level binary logistic regression model was conducted using SPSS 21.0. The coefficients and *p* values for each variable and interaction are shown in Table 2.

**Table 2:** The result of the two-level binary logistic regression model

Variables	Coefficient	*F*
Participant type	− 0.03	0.01
Age	− 0.15	1.33
BSSD facial manipulation	− 0.50***	10.49
WSSD facial manipulation	− 0.09	2.38
Non-facial cue	0.39	1.92
Participants type × BSSD facial manipulation	− 0.48	1.08
Participants type × WSSD facial manipulation	0.91*	3.79
Participant type × non-facial cue	− 0.26	0.49
Intercept (fixed effect)	− 0.73	
Intercept (random effect)	2.40***	
Model fit		
− 2LL	4116.15	
AIC	4118.16	
*F*	10.88***	

− 2LL = − 2 log likelihood; AIC = Akaike’s information criterion. Lower − 2LL and AIC indices indicate better model fit, overall. *p < .05, **p < .01, ***p < .001

The results of the two-level binary logistic regression analysis revealed a significant effect of facial manipulation method as well as a significant interaction between participant type and facial manipulation method. Therefore, a subsequent analysis compared face preferences between nonpregnant and pregnant women by facial manipulation method, and as the effect of non-facial cues was not significant, the two conditions for non-facial cues were combined in the following post hoc tests. The Bonferroni correction is applicable for controlling the experiment-wise error rate (EER) in multiple testing of post hoc comparisons, especially if the number of tests less than 5 (Bender & Lange, 2001). Therefore, we used a corrected *α*, which was the traditional significance level (*α* = .05), divided by the number of comparisons (Bender & Lange, 2001). For the present analysis, because we compared participants’ preference between three conditions, the corrected *α* was .05/3 (.017).

The results of the Mann–Whitney U tests indicated a significant effect of participant type only in the BSSD facial manipulation condition (*Z* = − 3.10, *p* = .002), and pregnant women presented a stronger preference (68.3%) for feminized male faces compared to nonpregnant women (46.4%). However, there was no preference difference between nonpregnant and pregnant women in the WSSD facial manipulation condition (*Z* = − 1.260, *p* = .208) and unmanipulated face conditions (*Z* = − 1.80, *p* = .072).

Friedman tests were used to examine the effect of facial manipulation method on nonpregnant and pregnant women and a significant effect was found among nonpregnant women, *χ*^*2*^ = 78.40, *p* < .001, rather than among pregnant women, *χ*^*2*^ = 2.21, *p* = .331. Further, Wilcoxon tests were used to compare face preferences between the three conditions in nonpregnant women with a corrected Bonferroni *α *= .017. The result demonstrated that nonpregnant women had the strongest preference for feminine male faces in the WSSD facial manipulation condition (83.6%, *Z* = − 4.33, *p* < .001), while they showed the least preference for feminine male faces in the BSSD facial manipulation condition (46.4%, *Z* = − 4.64, *p* < .001), as compared to the unmanipulated facial condition (66.8%).

## Discussion

This study aimed to examine the influence of pregnancy on Chinese women’s preference for sexual dimorphism in male faces. The results indicated that both pregnant and nonpregnant women preferred feminized male faces. Pregnant women showed a stable preference for feminized male faces in all three face manipulation conditions, while nonpregnant women’s preference for feminized male faces was influenced by face manipulation conditions and was insignificant in the BSSD face manipulation condition. These results provide new evidence of how hormones influence women’s preference for sexual dimorphism in male faces.

Consistent with previous research (Wen & Zuo, 2012), women in general preferred feminized male faces, regardless of participants’ pregnancy status. As suggested by the ecological system theory, the factors at the macro-level potentially influence the socialization processes at the micro-level, e.g., the individual preferences (Bronfenbrenner, 1977). China is a typical collectivistic society that advocates group benefits above individual gain (Wang & Cui, 2007). In Chinese culture, people are encouraged to be more modest, gentle, and polite, and emphasize interpersonal harmony (Wang & Cui, 2007). The influence of culture could increase the tolerance for the feminized appearance of males in China. One study compared adolescents’ preferences for masculine and feminine appearances in their peers in Hong Kong and America. The results indicated that the Chinese people adopted more tolerant attitudes toward feminized males, and this finding is consistent with the higher proportion of feminized male models exhibited in Chinese media (Gutierrez et al., 2020). Combined with the findings in this study, we infer that the “femininity” characteristics of culture could also manifest themselves in people’s preference for feminized male faces.

Pregnant women showed a stable preference for feminized male faces across different facial manipulations. This result may be due to the match between women’s perception of feminized male faces and pregnant women’s psychological needs (Perrett et al., 1998; Rhodes et al., 2005). The relationship between sexually dimorphic facial features, genetic benefits, and nourishing of offspring has been demonstrated in the good genes model (Fink & Penton-Voak, 2002; Gangestad & Simpson, 2000; Scott et al., 2014). Compared to nonpregnant women, pregnant women might place a greater emphasis on a male’s potential parental investment and be more sensitive to this factor in male faces. In addition, women believe that feminized male faces imply that the males will assume responsibility for nourishing offspring, provide financially for the family, and provide other factors beneficial for the survival of their next generation (Rhodes et al., 2005). This finding is consistent with the stable feminized preferences observed in this experiment in all three face manipulation conditions.

Our results also demonstrated the influence of the manipulation methods on women’s preferences. In the BSSD facial manipulation condition, pregnant women displayed a significant femininity preference in male faces that was not found in nonpregnant women. This finding suggests that compared to WSSD facial manipulation, BSSD facial manipulation has a higher sensitivity for distinguishing pregnant and nonpregnant women’s face preferences. This disparity may have resulted from the different logic underlying the facial manipulation methods. Masculine and feminine male faces in WSSD facial manipulation were obtained by averaging male faces higher and lower in masculinity ratings. However, for BSSD facial manipulation, face stimuli were generated by exaggerating or diminishing differences in the male and female prototypes. Compared to WSSD, BSSD has a wider range and higher sensitivity and potentially includes more feminized information to which pregnant women are more sensitive (Dixson et al., 2019). Therefore, a preference difference between pregnant women and nonpregnant women was observed in the BSSD facial manipulation condition.

In addition, this study found no significant influence of non-facial cues on women’s preferences for dimorphic faces, which is inconsistent with previous findings (e.g., Dixson et al., 2019; Dixson, Lee, Blake, Jasienska, & Marcinkowska, 2018; Neave & Shields, 2008). The existence of non-facial cues significantly influences the perceived masculinity ratings of male faces, and the influence of culture and face stimuli should be considered as potential reasons for the inconsistency we found. Compared to Western cultures, Chinese culture advocates a more feminized male appearance (Gutierrez et al., 2020), and photographs of college students might not display as many gender markers as faces collected from middle-aged individuals. Further research should explore the influence of culture and face stimuli to provide a more complete understanding of women’s perceptions of male faces.

### Limitations

Our study had some limitations. The age range of pregnant and nonpregnant women was not standardized, although the effect of age was insignificant in this analysis. We did not consider the influence of other demographic variables (e.g., menstrual cycle status and relationship status in nonpregnant women) in this research. Thus, future research should examine women’s preferences across different age ranges and explore how other demographic variables influence women’s preferences in male faces.

The photographs of men in this research were selected from a university database, which may have resulted in different results than those based on completely mature male faces (Othmani, Taleb, Abdelkawy, & Hadid, 2020; Wu & Wang, 2019). Future research should obtain face stimuli from diverse resources to increase the ecological validity of facial attractiveness research.

This research focused on women’s self-report responses regarding their preferences in male faces. Future research can examine biological changes, such as hormone fluctuations or brain activity, to understand the underlying mechanisms and potential psychological processes of women’s preference patterns. By combining behavioral and biological data, we can gain a better understanding of the psychophysiological mechanism of women’s preferences in male faces and how various indicators interact and influence this process.

Most previous research in this area was conducted in Western cultures, while this study explored women’s preferences for sexual dimorphism in male faces in Chinese culture. A direct cross-cultural comparison of women’s preferences for dimorphism in male faces is an important direction for future study.

### Conclusion

We investigated Chinese nonpregnant and pregnant women’s preferences for sexual dimorphism in male faces using three facial manipulation methods. We found a general preference for feminized male faces in Chinese culture; pregnant women showed a stable preference for femininity in the BSSD facial manipulation condition, while nonpregnant women’s preference for feminized male faces varied by face manipulation method. These results suggest the influence of women’s hormones on their preferences in male faces. Our findings support the trade-off model of strategic pluralism and enrich the understanding of evolutionary psychology.

## Electronic supplementary material

Below is the link to the electronic supplementary material.

Supplementary material 1 (DOCX 20 kb)
