# Supplementary material for: Non-Pregnant and Pregnant Women’s Femininity Preferences in Male Faces: Tests Based on Within- and Between-Sex Sexual Dimorphism Facial Manipulations
Source: Arch Sex Behav. 2021 Jan 4;50(2):531–41. doi: 10.1007/s10508-020-01868-8 (PMC7889572; doi:10.1007/s10508-020-01868-8)
Supplement: Supplementary file 1 — Supplementary material 1 (DOCX 20 kb) [file 10508_2020_1868_MOESM1_ESM.docx]

**Supplemental Materials**

**Manipulation check procedure**

To measure the effectiveness of face stimuli pairings, a manipulation check procedure was conducted. A total of 49 females were recruited online (*M* = 21.02, *SD* = 2.20), ranging in age from 18 to 28 years. Consistent with the formal experiment procedure, the unmasked face pairing for the three conditions was presented to each participant, who was required to choose the more masculinized face from each pairing and rate its level of masculinity (from 1 = a little more masculine compare to the other, to 4 = far more masculine compare to the other). The result of this manipulation check procedure is shown in Table S1.

The choice ratio indicated the percentage of participants who chose the relatively more masculine face in each pairing. For example, in the F-M pairing, it was the masculinized face; in the F-N pairing, it was the neutralized face; in the N-M pairing, it was the masculinized face. From this table, we see that in most circumstances, more than 79.6% of participants could distinguish the more masculinized face in each pairing, except for the N-M pairing in the BSSD facial manipulation condition (46.9%). However, the total choice ratio in the BSSD facial manipulation condition was 74.1%, which indicated an overall acceptable reliability of facial measurement in the BSSD facial manipulation condition.

In addition, participants’ ratings for the masculinity level of the face they chose from each pairing is also shown in this table. For participants who perceived the more feminized male face as more masculine, their ratings for feminized faces were averaged as Rating-feminized face. In contrast, for participants who perceived the more masculinized male face as more masculine, their ratings for masculinized faces were averaged as Rating-masculinized face. Considering the imbalanced number of participants’ choices in most circumstances, we used Cohen’s *d* to evaluate the distinctions of the two faces that appeared in each pairing. A larger Cohen’s *d* indicated larger distinctions between two faces. As shown in the table, a small to large distinction was detected in all circumstances, Cohen’s *d* = [0.11, 1.28].

Therefore, in most circumstances, participants were able to recognize the more masculine face in each pairing and can distinguish two faces presented at the same time.

**Table S1.**

*Participants’ masculine male choices ratio and relative masculinity rating for all face pairings.*

| Condition | Face pairing | Choice ratio | Rating-feminized face *M*(*SD*) | Rating-masculinized face *M*(*SD*) | Cohen’s *d* |
| --- | --- | --- | --- | --- | --- |
| BSSD | F-M | 85.7 | 2.57(1.27) | 2.93(0.89) | 0.38 |
|  | F-N | 89.8 | 3.20(1.30) | 2.86(0.91) | 0.36 |
|  | N-M | 46.9 | 2.62(0.80) | 2.78(1.09) | 0.17 |
| Total effect | | 74.1 | 2.68(0.96) | 2.87(0.93) | 0.20 |
| WSSD | F-M | 89.8 | 2.00(0.71) | 3.07(0.85) | 1.28 |
|  | F-N | 79.6 | 2.60(0.70) | 2.95(0.89) | 0.41 |
|  | N-M | 83.7 | 3.00(0.93) | 2.78(1.01) | 0.22 |
| Total effect | | 84.4 | 2.61(0.84) | 2.94(0.92) | 0.36 |
| Unmanipulated faces | F-M | 91.8 | 2.25(0.96) | 3.18(0.94) | 0.99 |
|  | F-N | 83.7 | 3.00(0.82) | 2.62(1.05) | 0.15 |
|  | N-M | 91.8 | 2.75(0.89) | 2.59(1.10) | 0.37 |
| Total effect | | 89.1 | 2.69(0.87) | 2.80(1.06) | 0.11 |
